# No association of the insulin gene VNTR polymorphism with polycystic ovary syndrome in a Han Chinese population

**DOI:** 10.1186/1477-7827-7-141

**Published:** 2009-12-01

**Authors:** Yuping Xu, Zhaolian Wei, Zhiguo Zhang, Qiong Xing, Pin Hu, Xiaohui Zhang, Guihua Gao, Yong Wang, Qian Gao, Long Yi, Yunxia Cao

**Affiliations:** 1 Department of Obstetrics & Gynecology, the First Affiliated Hospital, Anhui Medical University, Hefei, 230022, China; 2 Department of Pathology, Nanjing University Medical School, Nanjing, 210093, China

## Abstract

**Background:**

Polycystic ovary syndrome (PCOS) is a common endocrine disorder associated with an increased risk of type II diabetes mellitus. The results of previous research about the association of the VNTR polymorphism in 5-prime flanking region of the insulin (INS) gene with PCOS have been inconsistent. The present study was to investigate the association of the INS-VNTR polymorphism with PCOS in a Han Chinese population.

**Methods:**

The -23/HphI polymorphism as a surrogate marker of the INS-VNTR length polymorphism was genotyped by polymerase chain reaction and restriction fragment length polymorphism (PCR-RFLP) in 216 PCOS patients and 192 non-PCOS women as a control group. Allelic and genotypic frequencies were compared between patients and controls, and these results were analyzed in respect to clinical test data.

**Results:**

No significant differences were observed between the cases and controls groups either in allele (P = 0.996) or genotype (P = 0.802) frequencies of INS-VNTR polymorphism; Regarding anthropometric data and hormone levels, there were no significant differences between INS-VNTR genotypes in the PCOS group, as well as in the non-PCOS group.

**Conclusion:**

The present study demonstrated for the first time that the INS-VNTR polymorphism is not a key risk factor for sporadic PCOS in the Han Chinese women. Further studies are needed to give a global view of this polymorphism in pathogenesis of PCOS in a large-scale sample, family-based association design or well-defined subgroups of PCOS.

## Background

Polycystic ovary syndrome (PCOS) is a common endocrine disorder affecting up to 4% of women of reproductive age. The disorder is characterized by hyperandrogenism, menstrual irregularities and often central obesity. Moreover, it is associated with an increased risk of type II diabetes mellitus [1]. Colilla et al. demonstrated that there is a heritable component in beta-cell dysfunction in families of women with PCOS, and that heritability of beta-cell dysfunction is likely to be a significant factor in the predisposition to diabetes in PCOS [2].

The insulin (INS) gene locates at chromosome 11p15.5 and is one of established susceptibility locus to type II diabetes in Caucasians [3]. In the 5-prime flanking region of the INS gene, a variable number of tandem repeat (VNTR) regulates transcription of the gene; the shortest (class I) alleles were found to increase, whereas the longest (class III) alleles were observed to decrease in the patients in comparison to the controls [4,5]. It was reported that most class III alleles are associated with higher levels of INS transcription than class I alleles in the thymus [6,7]. The higher INS expression may more efficiently induce tolerance to insulin. An A/T single nucleotide polymorphism (SNP) located at -23 bp is highly linked with the INS-VNTR length polymorphism. The SNP can be digested by the restriction endonuclease HphI and exhibits restriction fragment length polymorphism which is called "-23/Hph I polymorphism" as a surrogate marker of the INS-VNTR length polymorphism [8].

The association between the polymorphism and PCOS has been observed in Caucasians but the results were inconsistent. Waterworth et al were the first to provide evidence on a linkage of PCOS with the INS gene VNTR locus and an association between VNTR class III alleles and the subset of anovulatory PCOS subjects [9]. A study of 74 UK women with PCOS reported an association between the class III allele and increasing severity of clinical phenotype [10]. On the contrary, three independent research groups failed to replicate the association [11-13]. On account of these controversial reports, the association has to be confirmed by independent studies in different ethnic groups. The purpose of the present study is to examine the genetic association of the -23/Hph I polymorphism in the INS gene with PCOS in a Han Chinese population.

## Methods

### Subject

The subjects were recruited from patients who visited the Department of Obstetrics and Gynecology of the First Affiliated Hospital of Anhui Medical University during December 2005 and December 2006, including 216 PCOS patients and 192 non-PCOS women as a control group. The inclusion criteria of the PCOS group was based on the Rotterdam diagnostic criteria [14], while the eligibility criteria of non-PCOS women included: menstrual period < 35 d (most women had been pregnant or given birth to at least one child), precluding obesity, hirsutism, acne, excess sebum production and insulin resistance. All subjects were ethnic Han Chinese living in Anhui province and had not received hormonal therapy for at least 3 months before hormonal assays. After informed consent was obtained, blood was drawn from the subjects for hormonal assays and DNA analysis under the supervision of the Ethical Committee of the First Affiliated Hospital, Anhui Medical University. The peripheral blood was obtained between 8 AM and 9 AM after a 12-hour overnight fast during the 3rd to the 5th day of the menstrual cycle.

### Hormonal assays

The level of serum follicle-stimulating hormone (FSH), luteinizing hormone (LH), total testosterone (T), estrogen (E2) and prolactin (PRL) were determined using Commercially available human chemiluminescence enzyme immunoassay kits (ROCHE DIAGNOSTICS GMBH, Mannheim, Germany). The intra- and inter-assay coefficients of variation of all the assays were less than 10%.

### Genotyping

**Figure 1:**
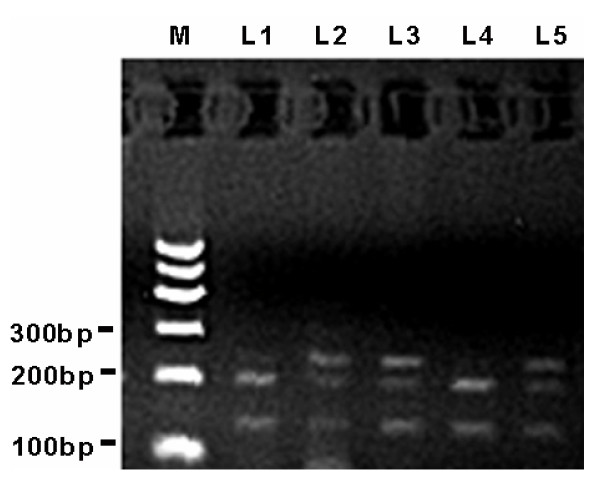
**Representative allele patterns of genotyping for INS-23Hph I polymorphism on 2.5% agarose gel**. M: DNA marker; L1 and L4: bands of 191 plus 129 bp indicating genotype AA; L2, L3 and L5: bands of 231 and 191 plus 129 bp indicating genotype AT;

Genomic DNA was prepared from peripheral blood leukocytes by using the Chelex-100 method [15]. INS-23/Hph I polymorphism was determined using the polymerase chain reaction and restriction fragment length polymorphism (PCR-RFLP) method [12]. The PCR reaction mixture contained 50 ng genomic DNA, 0.5 μM of each primer (forward: 5'-AGC AGG TCT GTT CCA AGG-3' and reverse: 5'-CTT GGG TGT GTA GAA GAA GC-3'), 10 mM Tris-HCl (pH 9.0), 50 mM KCl, 1.5 mM MgCl2, 0.1% Triton X-100, 0.2 mM of each dNTP, and 0.75 unit Tag DNA polymerase (Promega, USA) in a final volume of 25 ml. The reaction mixture was subjected to denaturation at 96°C for 2 min, followed by 30 cycles at 94°C for 1 min, 62°C for 1 min, 72°C for 1 min, then by a final extension at 72°C for 10 min. The PCR products were 360 bp and were digested with the Hph I restriction enzyme at 37°C for about 18 h. After digested products were electrophoresed on a 2.5% agarose gel and visualized by ethidium bromide staining, the T and A alleles could be distinguished as bands of 231 plus 129 bp and 191 plus 129 bp, respectively. Representative allele patterns of genotyping are illustrated in Figure 1.

### Statistical analysis

The standard *X*^2 ^tests were used to compare allele or genotype frequencies in PCOS and non-PCOS groups and among different ethnic groups, and verify the Hardy-Weinberg equilibrium of genotype frequencies. The results of serum hormone levels were reported as MEANS ± SD. Differences in anthropometric data and serum hormone levels between PCOS and non-PCOS groups were assessed by using Student's *t*-test. P < 0.05 was considered significant for all tests. Statistical analyses were performed using the SPSS statistical software (SPSS Inc., Chicago, IL).

## Results

Table 1 shows comparison of anthropometric data and hormone levels between PCOS and non-PCOS groups (some subjects whose hormone measurement is different from that presented in the Materials and Methods have been excluded). There is no significant difference in menarche age, FSH and PRL between the two groups. The BMI, serum LH, LH/FSH and serum T of the PCOS group are significantly higher than those of the non-PCOS group, which is consistent with the clinical characteristics of PCOS [14]. However, the level of E2 was also significantly higher in the PCOS group compared to the control. The unexpected result cannot be explained easily and further studies may be needed to address the issue.

**Table 1:** Anthropometric data and hormone levels of PCOS and non-PCOS groups

Group	N	Age(years)	Menarche age (years)	BMI (kg/m^2^)	FSH (IU/ml)	LH (IU/ml)	LH/FSH	T (nmol/L)	E2 (pmol/L)	PRL (ng/ml)
PCOS	190	26.81 ± 4.25*	14.49 ± 1.62	23.08 ± 3.65*	6.38 ± 4.37	14.06 ± 8.38*	2.40 ± 1.27*	2.30 ± 1.09*	228.32 ± 166.28*	15.35 ± 8.53
Non-PCOS	157	31.53 ± 4.31	14.66 ± 2.41	21.27 ± 2.24	6.97 ± 2.08	4.46 ± 2.10	0.66 ± 0.32	1.65 ± 3.92	179.75 ± 143.00	18.42 ± 20.71

Note: *P < 0.05 vs non-PCOS. Some subjects whose hormone measurement is different from that presented in the Materials and Methods have been excluded.

The allele and genotype distributions for both PCOS and non-PCOS groups were consistent with the Hardy-Weinberg equilibrium, and are shown in Table 2. Frequencies of the allele T and genotype AT plus TT of non-PCOS (7.3% and 14%, respectively) were significantly different to those previously published in the Japan population (3.3% and 6.5%, respectively, *P *< 0.05) [16] and UK Caucasians (27.9 and 47.4, respectively, *P *< 0.05) [13]. In the association study, no significant difference between cases and controls was observed either in allele (*P *= 0.996) or in genotype (*P *= 0.802) frequencies, indicating that there was no association between INS-23/Hph I polymorphism and the incidence of sporadic PCOS cases in the Han Chinese population.

**Table 2:** Allelic and genotypic frequencies of INS-23/Hph I polymorphism

	Allele n (%)			Genotype n (%)			
Subjects	A	T	P	A//A	A//T	T//T	*P*
PCOS	407 (94.2)	25 (5.8)	0.996	193 (89.4)	21 (9.7)	2 (0.9)	0.802
Non PCOS	356 (92.7)	28 (7.3)		165 (85.9)	26 (13.5)	1 (0.5)	

As indicated in Table 3, there was no significant difference regarding anthropometric data and hormone levels between INS-23/Hph I genotypes in the PCOS group and those in the non-PCOS group.

**Table 3:** Anthropometric data and hormone levels of different INS genotypes in the PCOS and the non-PCOS groups

	PCOS			Non PCOS		
Index	A/A	A/T+ T/T	P	A//A	A/T+ T/T	P
N (%)	173 (91.05)	17 (8.95)	-	134 (85.35)	23 (14.65)	-
Age (years)	26.69 ± 4.28	27.94 ± 3.93	0.249	31.66 ± 4.27	30.74 ± 4.60	0.344
Menarch Age (years)	14.46 ± 1.58	14.88 ± 2.00	0.301	14.67 ± 2.52	14.57 ± 1.65	0.846
BMI (kg/m^2^)	22.96 ± 3.55	24.30 ± 4.52	0.149	21.23 ± 2.16	21.49 ± 2.68	0.610
FSH (IU/ml)	6.43 ± 4.55	5.88 ± 1.84	0.625	6.96 ± 2.17	7.05 ± 1.44	0.851
LH (IU/ml)	14.30 ± 8.64	11.68 ± 4.64	0.221	4.43 ± 2.09	4.64 ± 2.23	0.670
LH/FSH	2.47 ± 1.29	2.12 ± 0.95	0.347	0.66 ± 0.31	0.68 ± 0.36	0.155
T (nmol/L)	2.33 ± 1.29	1.99 ± 0.87	0.228	1.73 ± 4.22	1.24 ± 0.89	0.587
E2(pmol/L)	225.11 ± 163.90	260.93 ± 191.33	0.398	179.79 ± 139.70	179.53 ± 164.35	0.994
PRL(ng/ml)	16.23 ± 12.15	16. 97 ± 8.31	0.394	16.97 ± 9.56	26.87 ± 49.01	0.345

Note: some subjects whose hormone measurement is different from that presented in the Materials and Methods have been excluded.

## Discussion

The association of the VNTR polymorphism in 5-prime flanking region of the INS gene susceptible to type II diabetes with PCOS have been studied over the years in different populations, and the results observed have been inconsistent and controversial. No such data is available in the Han Chinese population. In the present study we have analyzed for the first time the association between the polymorphism of PCOS and anthropometric data and hormone levels.

Some of PCOS phenotypes have been linked to the INS-VNTR. Waterworth et al. were the first to provide the evidence on an association between VNTR class III alleles and the subset of anovulatory and hyperinsulinaemic PCOS subjects in a recessive genetic model [9]. Ferk et al. also found that Class III INS-VNTR alleles were significantly more frequent in the Slovene PCOS patients and the interaction of obesity and the III/III INS VNTR genotype might be a risk factor in the development of PCOS [17].

In this study, we demonstrate that there is no association between INS-23/Hph I polymorphism and PCOS in the Han Chinese population. Moreover, further analysis showed that there was no significant difference regarding anthropometric data and hormone levels between INS-VNTR genotypes in the PCOS group, as well as in the non-PCOS group. The results were consistent to those previously reported [11-13]. After investigating 96 hyperandrogenic patients and 38 healthy control women, Calvo et al. concluded that there is no association between INS-VNTR and PCOS or hyperandrogenism, at least in Spanish women [12]. Subsequently, in a large-scale research on 255 nuclear families and about 3000 subjects from two populations (Britisher/Irisher and Finlander), Powell et al. revealed that INS-VNTR was not a key factor in the pathogenesis and progress of PCOS [13]. It should be noted that studies to date failed to confirm a significant association with the PCOS in general.

There are several possible explanations for these inconsistent results of association between the INS-VNTR and PCOS. Previous studies including this one showed the allele and genotype of the VNTR polymorphism in INS gene were different among ethical populations. Osawa et al. found the A allele frequency of -23/HphI polymorphism was 97.4% in Japanese subjects, whereas in Europeans it was about 30% [18]. The results in this study showed the A allele frequency was about 93.5% (Table 2). Because of the predominance of class I over class III alleles in Asian population, further study with large-scale sample design is needed for us to ascertain the association between the INS-VNTR and PCOS. Case-control study is a common and powerful design to detect genetic contributions to complex diseases like PCOS. Unfortunately, a small portion of differences in allele frequencies between cases and controls could be attributable to diversity in background population, which is called population stratification. Family-based association designs like transmission disequilibrium test measure the over-transmission of an allele from heterozygous parents to affected offsprings. The designs do not use control groups and can overcome this disadvantage effectively [19], and would be an alternative way to assess the risk of PCOS.

In this study, we did not assess Insulin levels and Insulin resistance of the subjects. We will address the issue of whether subgroups of PCOS are associated with INS-VNTR polymorphism by investigating clinical and biochemical features related to glucose metabolism among PCOS women.

## Conclusion

The present study demonstrated for the first time that the INS-VNTR polymorphism is not a key risk factor in sporadic PCOS in the Han Chinese women. Further studies are needed to give a global view of this polymorphism in pathogenesis of PCOS in a large-scale sample, family-based association design or well-defined subgroups of PCOS.

## Competing interests

The authors declare that they have no competing interests.

## Authors' contributions

YC and LY conceived and designed the study. LY, QG and YW contributed the data analysis and drafted the manuscript. YX, ZW and ZZ carried out the molecular genetic studies. QY, PH and carried out the immunoassays. XZ and GG performed collection of blood samples. All authors read and approved the final manuscript.
